# Persistent iatrogenic atrial septal defect after single-puncture, double transseptal access for 4D ICE-guided left atrial appendage occlusion: Incidence and clinical outcomes

**DOI:** 10.1007/s10840-026-02440-5

**Published:** 2026-09-16

**Authors:** Kasen L Culler, Aamir Ahmed, Supreet Singh, Graham Peigh, Korosh Sharain, Aishwarya Pastapur, Aravind Kalluri, Anirudh Nandan, Chad Gier, Erik Simon, Alexandru B Chicos, Kaustubha Patil, Anna Pfenniger, Albert C Lin, Rod S Passman, Bradley P Knight, Susan Kim, Nishant Verma

**Affiliations:** Division of Cardiology, Northwestern University Feinberg School of Medicine, Chicago, IL USA

**Keywords:** Persistent atrial septal defect, Left atrial appendage occlusion, Intracardiac echocardiography, Transseptal puncture

## Abstract

**Background:**

Persistent iatrogenic atrial septal defect (iASD) is a known consequence of transseptal puncture (TSP). Large-bore double transseptal access through a single TSP is commonly used for intracardiac echocardiography (ICE)-guided left atrial appendage occlusion (LAAO). This study evaluated the incidence and clinical outcomes of persistent iASD following this approach.

**Methods:**

We retrospectively reviewed LAAO procedures at a single academic center from January 2020 to December 2024. Cases were grouped as: (1) 4D ICE with double transseptal access (*n* = 58), (2) 2D ICE with double transseptal access (*n* = 64), or (3) no left atrial (LA) ICE (*n* = 81). Patients were included if they had follow-up imaging (TEE or cardiac CT). Multivariable logistic regression identified predictors of persistent iASD.

**Results:**

Among 203 patients (age 77.1 ± 6.6 years; 59% male), follow-up occurred at 58.0 ± 25.2 days. Persistent iASD was detected in 88 patients (43%). ICE-guided cases had a higher incidence of iASD compared with no LA ICE (54.1% vs. 27.2%; *p* < 0.001), with no difference between 2D and 4D ICE (*p* = 0.340). On multivariable analysis, both 2D and 4D ICE were independently associated with increased odds of persistent iASD (2D ICE: OR 3.45, 95% CI 1.46–8.17; 4D ICE: OR 3.57, 95% CI 1.52–8.38). Coronary artery disease was associated with increased odds (OR 2.44, 95% CI 1.12–5.28), while hypertension was associated with decreased odds (OR 0.41, 95% CI 0.18–0.96). Persistent iASD was not associated with adverse clinical outcomes.

**Conclusions:**

Persistent iASD is common following LAAO, occurring in 43% of patients at early follow-up. Left atrial ICE use is independently associated with increased odds of persistent iASD, with similar rates between 2D and 4D ICE techniques.

## Background

Atrial fibrillation (AF) is the most common cause of cardioembolic stroke [1], making stroke prevention one of the most important considerations in AF management [2]. Oral anticoagulants (OACs) are effective in reducing the risk of embolism, but are contraindicated in some patients due to a high risk of adverse bleeding events [3] or suboptimal daily compliance [4]. Since the majority of AF-related cardiac thrombi are identified in the left atrial appendage [5], left atrial appendage occlusion (LAAO) has emerged as a useful and noninferior alternative method of stroke prevention in nonvalvular AF [2, 6].

LAAO involves deployment of an occlusion device in the left atrial appendage through a transseptal sheath. Accurate imaging is critical to procedural success and safety, and transesophageal echocardiography (TEE) has historically served as the standard intraprocedural imaging modality used to guide LAAO device deployment. However, in recent years, the use of intracardiac echocardiography (ICE) during LAAO procedures has been increasingly adopted. Traditional two-dimensional (2D) ICE was initially used in LAAO procedures to provide real-time intracardiac visualization while avoiding the need for general anesthesia. More recently, four-dimensional (4D) ICE has been introduced, offering a wider field of view and multiplanar reconstruction, which may improve visualization of complex left atrial appendage anatomy and device positioning^7^. The use of ICE to guide LAAO typically requires advancement of the imaging catheter across the interatrial septum and into the LA. To achieve this, operators often utilize double transseptal access through a single transseptal puncture [7]. This requires additional dilation of the puncture site to accommodate both the device sheath and the imaging catheter, which may result in greater mechanical stretch of the interatrial septum.

Iatrogenic atrial septal defects (iASD) are potential complications of any catheter-based procedure requiring transseptal puncture. They may close spontaneously or persist and have been associated with quantifiable hemodynamic changes in some patients [8–10]. These defects may also cause clinical complications related to interatrial shunting such as hypoxemia or exertional dyspnea [11, 12]. Although some data has been reported regarding the rate of iASD persistence after LAAO [13–17], existing studies primarily involve procedures utilizing 2D ICE or TEE and do not specifically evaluate the complications of single-puncture double transseptal access in the era of 4D ICE-guided LAAO. Therefore, the impact of additional septal dilation with the larger bore 4D ICE catheter and iASD has not previously been explored.

This study aims to evaluate the incidence and clinical outcomes of persistent iASD following single-puncture double transseptal access during LAAO procedures, comparing cases utilizing 4D ICE, 2D ICE and TEE without LA ICE.

## Methods

### Study design

This was a single-center, retrospective observational cohort study of patients who underwent LAAO at a single academic medical center (Northwestern Memorial Hospital) between January 1, 2020 and December 31, 2024. The study was approved by our institutional review board. All consecutive patients who underwent LAAO with a Watchman or Amulet device during the study period were screened for inclusion.

### Study population

Patients were eligible for inclusion if they: (1) were 18 years of age or older, (2) had a diagnosis of non-valvular AF and (3) had follow-up imaging with either transesophageal echocardiography (TEE) or cardiac computed tomography (CT) within 6 months of LAAO. Patients were excluded if: (1) at least 45 days of follow up data was not available at the time of data collection or (2) they had known pre-existing patent foramen ovale or inter-atrial shunt.

Included patients were divided into 3 comparison groups based on ICE imaging modality used during LAAO: (1) 4D ICE with double transseptal access through a single transseptal puncture (TSP), (2) 2D ICE with double transseptal access through a single TSP, or (3) no left atrial ICE catheter.

### LAAO procedure and transseptal technique

LAAO procedures were performed in a dedicated electrophysiology laboratory by experienced operators according to institutional practice. LAAO device size was selected based on TEE and angiographic measurement of the LAA. Imaging modality for procedural guidance was chosen based on available technology and operator preference. All LAAO procedures, with or without ICE, included the use of traditional transesophageal echocardiography for intraprocedural imaging.

Ultrasound-guided femoral venous access was obtained in all cases. In procedures utilizing 4D ICE, a 10 F intracardiac echocardiography catheter was introduced via an 11 F sheath in the left common femoral vein. Procedures involving 2D ICE in the LA were performed in the same manner, except a 9 F short sheath was placed in the left common femoral vein to accommodate an 8 F 2D ICE catheter. A 14–17 F LAAO delivery sheath was advanced into the right atrium via the right femoral vein. Transseptal access was obtained using a radiofrequency wire under echocardiographic and fluoroscopic guidance. In cases involving left atrial ICE, a single transseptal puncture was used to facilitate double transseptal access, allowing both the delivery sheath and ICE catheter to be advanced across the interatrial septum. Septal dilation was performed using sheath-mediated dilation alone, without the use of balloon dilation. The above technique for obtaining transseptal access was standardized across operators.

LAAO procedures without any ICE catheter in the LA were performed similarly, though without the interatrial septum dilation. In these cases, an ICE catheter was maintained in the right atrium for adjunctive visualization but was not advanced across the interatrial septum.

### Primary outcome and imaging parameters

The primary outcome in this study was the presence of persistent iASD on follow-up imaging after LAAO. All patients underwent follow up TEE or CT between 1 and 6 months after device placement. iASD was defined as a residual interatrial communication at the site of prior transseptal puncture. Defects consistent with patent foramen ovale (PFO), including with a flap-valve or tunnel-like morphology, were not classified as iASD.

Comprehensive transthoracic and transesophageal echocardiography was performed according to current recommendations and guidelines. Both were performed with commercially available echocardiographic systems and probes allowing visualization of the inter-atrial septum in at least two perpendicular planes with and without color Doppler.

Independent review was performed by a fellowship-trained cardiac imaging specialist to assess for the presence or absence of persistent iASD. On TEE, the presence of iASD was assessed using color Doppler as well as saline contrast with and without Valsalva. Shunt direction was recorded for patients with TEE follow up. On CT, direct visualization and contrast evaluation was utilized to assess the presence of iASD. Shunt direction was recorded for patients with CT follow up when deemed feasible based on quality of contrast images.

Readers were not blinded to procedural group due to the retrospective nature of the study.

### Additional follow up

All patients were scheduled for follow up visits at 3, 6 and 12 months after LAAO. The primary endpoint of persistent iASD was determined using the first protocol follow-up TEE or cardiac CT performed between 1 and 6 months after LAAO. Although patients underwent clinical follow-up through 12 months, subsequent echocardiographic studies performed for other clinical indications were rare, and were not systematically analyzed for iASD persistence.

Retrospective chart review was conducted by study personnel to monitor procedural results and changes in clinical status. Prescribed antiplatelet and antithrombotic therapies were monitored, as well as thromboembolic complications such as stroke, transient ischemic events, peripheral thromboembolism and bleeding complications based on documented clinic and telephone encounters available in the electronic medical record. Emergency department visits and hospitalizations within one year of LAAO were also recorded. Presence of these events was based on chart documentation. These clinical outcomes were collected as secondary safety outcomes and were analyzed descriptively rather than as a predefined composite endpoint.

### Statistical analysis

Univariate analyses were performed using χ^2^ tests for categorical variables and independent t-tests or analysis of variance (ANOVA) for continuous variables. For categorical variables, if any cell value was zero or had an expected frequency less than five, an exact test was utilized instead of a χ^2^ test.

A multivariable logistic regression model was constructed to identify independent predictors of persistent iASD at follow-up. Covariates included clinically relevant baseline characteristics including age, sex, coronary artery disease, hypertension and intraprocedural imaging modality. Imaging modality was modeled as a categorical variable with no left atrial ICE as the reference group. Adjusted odds ratios (ORs) with 95% confidence intervals were reported. A two-sided p-value < 0.05 was considered statistically significant. All statistical analysis was performed using SAS version 9.4 (SAS Institute Inc., Cary, NC). Follow-up imaging modality was not included in the multivariable model because it represents a post-procedural diagnostic assessment rather than a baseline or procedural characteristic and therefore was not considered an appropriate covariate for adjustment.

## Results

**Fig. 1:**
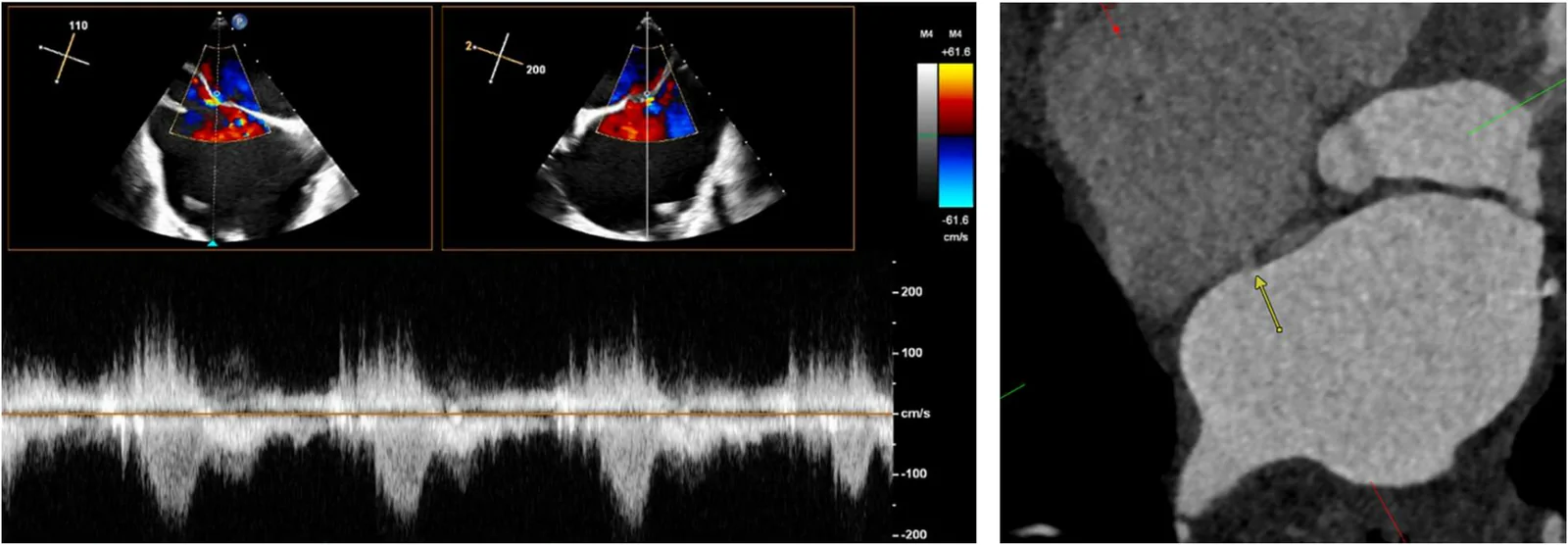
Representative imaging of persistent iatrogenic atrial septal defect (iASD)

A total of 203 patients who underwent LAAO and had follow-up imaging within 6 months were included in this analysis. The mean age of the cohort was 77 ± 7 years and 59% were male. Persistent iASD was identified on follow-up imaging in 88 patients (43.3%), while 115 patients (56.7%) had no evidence of persistent iASD. The presence of persistent iASD on follow-up imaging is illustrated in Fig. 1.

Figure 1: Left: Transesophageal echocardiography (TEE) with color Doppler demonstrating interatrial shunting across the iASD. Right: Cardiac computed tomography (CT) showing a defect in the interatrial septum consistent with a persistent iASD (yellow arrow).

### Baseline, procedural, and follow-up characteristics by imaging strategy

Baseline, procedural, and follow-up characteristics stratified by intraprocedural imaging modality are shown in Table 1. Overall, baseline characteristics were similar across groups. However, prior cerebrovascular accident (*p* = 0.027), chronic kidney disease (*p* = 0.043), post-procedural anticoagulation use (*p* = 0.010), and follow-up imaging modality (*p* = 0.001) differed significantly between groups.

**Table 1:** Baseline and procedural characteristics by intraprocedural imaging modality

	4D ICE (*n* = 58)	2D ICE (*n* = 64)	No LA ICE (*n* = 81)	*P*-Value
Baseline Characteristics				
Age, years	78 ± 7	76 ± 7	77 ± 6	
Male, *n* (%)	29 (50.0%)	40 (62.5%)	51 (50.0%)	0.247
Smoking, *n* (%)	3 (5.2%)	4 (6.3%)	4 (4.9%)	0.937
CHA_2_DS_2_-VASc, *n* (%)				0.936
1	0 (0.0%)	1 (1.6%)	0 (0.0%)	
2	3 (5.2%)	3 (4.7%)	5 (6.2%)	
3	13 (22.4%)	20 (31.3%)	25 (30.9%)	
≥4	42 (72.4%)	40 (62.5%)	51 (63.0%)	
Heart Failure, *n* (%)	17 (29.3%)	16 (25.0%)	31 (38.3%)	0.212
Coronary Artery Disease, *n* (%)	25 (43.1%)	22 (34.4%)	32 (39.5%)	0.608
Cerebrovascular Accident, *n* (%)	14 (24.1%)	18 (28.1%)	9 (11.1%)	0.027
Diabetes Mellitus, *n* (%)	21 (36.2%)	21 (32.8%)	24 (29.6%)	0.715
Hypertension, *n*(%)	41 (70.7%)	47 (73.4%)	65 (80.2%)	0.396
Thyroid Disease, *n* (%)	9 (15.5%)	14 (21.9%)	20 (24.7%)	0.421
Cancer, *n* (%)	9 (15.5%)	15 (23.4%)	17 (21.0%)	0.539
Chronic Kidney Disease, *n* (%)	12 (20.7%)	24 (37.5%)	17 (21.0%)	0.043
Prior PVI, *n*(%)	5 (8.6%)	9 (14.1%)	5 (6.2%)	0.262
Left Ventricular Ejection Fraction (%)	60 ± 11	57 ± 10	58 ± 10	0.236
Procedural Characteristics				
LA Dwell Time, minutes	58 ± 29	62 ± 17	61 ± 18	0.635
LAAO Device Size, mm	28 ± 4	27 ± 4	27 ± 3	0.008
Follow-up Characteristics				
Post-procedural Anticoagulation, *n* (%)	45 (77.6%)	45 (70.3%)	73 (90.1%)	0.010
Follow-up with TEE, *n* (%)	48 (82.8%)	63 (98.4%)	79 (97.5%)	0.001
Time to Follow-up Imaging, days	53 ± 14	63 ± 29	57 ± 28	0.076

Values are mean ± SD or *n* (%). *PVI* pulmonary vein isolation, *LV*  left ventricle, *LAAO* left atrial appendage occlusion

Continuous variables were compared using ANOVA and categorical variables using χ² or Fisher’s exact test

Among procedural variables, left atrial dwell time was similar across groups (*p* = 0.635), while LAAO device size differed modestly (*p* = 0.008). Time to follow-up imaging was also comparable between groups (*p* = 0.076).

### Univariate outcomes analysis

Baseline, procedural, and follow-up characteristics stratified by the presence or absence of persistent iASD are shown in Table 2. There was no significant difference in age (78 ± 7 vs. 77 ± 6 years, *p* = 0.263) or sex distribution (56.8% vs. 60.9% male, *p* = 0.561) between groups.

**Table 2:** Univariate analysis for persistent iASD

	Persistent iASD (*n* = 88)	No Persistent iASD (*n* = 115)	*P*-Value
Baseline Characteristics			
Age, years	78 ± 7	77 ± 6	
Male, *n* (%)	50 (56.8%)	70 (60.9%)	0.561
Smoking, *n* (%)	8 (9.1%)	3 (2.6%)	0.060
CHA_2_DS_2_-VASc, *n* (%)			0.150
1	0 (0.0%)	1 (0.9%)	
2	4 (4.5%)	7 (6.1%)	
3	19 (21.6%)	39 (33.9%)	
≥4	65 (73.9%)	68 (59.1%)	
Heart Failure, *n* (%)	33 (37.5%)	31 (27.0%)	0.109
Coronary Artery Disease, *n* (%)	43 (48.9%)	36 (31.3%)	0.011
Cerebrovascular Accident, *n* (%)	18 (20.5%)	23 (20.0%)	0.936
Diabetes Mellitus, *n* (%)	31 (35.2%)	35 (30.4%)	0.470
Hypertension, *n* (%)	59 (67.0%)	94 (81.7%)	0.016
Thyroid Disease, *n* (%)	13 (14.8%)	30 (26.1%)	0.051
Cancer, *n* (%)	19 (21.6%)	22 (19.1%)	0.665
Chronic Kidney Disease, *n* (%)	22 (25.0%)	31 (27.0%)	0.753
Prior PVI, *n* (%)	8 (9.1%)	11 (9.6%)	0.908
Left Ventricular Ejection Fraction (%)	58 ± 11	59 ± 9	0.412
Procedural Characteristics			
LA Dwell Time, minutes	59 ± 22	62 ± 21	0.477
LAAO Device Size, mm	27 ± 4	27 ± 4	0.502
Follow-up Characteristics			
Post-procedural Anticoagulation, *n* (%)	70 (79.5%)	93 (80.9%)	0.814
Follow-up with TEE, *n* (%)	80 (90.9%)	110 (95.7%)	0.171
Time to Follow-up Imaging, days	55 ± 21	60 ± 28	0.197

Values are mean ± SD or *n* (%). *PVI* pulmonary vein isolation, *LAAO*  left atrial appendage occlusion

Continuous variables were compared using Student’s t-test and categorical variables using χ² or Fisher’s exact test

Most clinical comorbidities, including heart failure, cerebrovascular accident, diabetes mellitus, cancer, chronic kidney disease, and prior pulmonary vein isolation, were similar between groups.

Coronary artery disease was more prevalent in patients with persistent iASD (48.9% vs. 31.3%, *p* = 0.011), whereas hypertension was more common in those without persistent iASD (81.7% vs. 67.0%, *p* = 0.016). Left ventricular ejection fraction and CHA₂DS₂-VASc score distribution were similar between groups.

Procedural and follow-up variables, including left atrial dwell time (*p* = 0.477), LAAO device size (*p* = 0.502), post-procedural anticoagulation (*p* = 0.814), and time to follow-up imaging (*p* = 0.197), did not differ significantly between groups. Major periprocedural complications were uncommon. Five major complications occurred overall, all in patients without persistent iASD (0.0% in patients with iASD vs. 4.3% in those without, *p* = 0.070). These included three pericardial effusions, one pneumothorax, and one major bleeding event. The pneumothorax was considered most likely related to anesthesia-associated barotrauma rather than the LAAO procedure itself.

### Incidence of persistent iASD by imaging strategy

The incidence of persistent iASD differed significantly according to intraprocedural imaging strategy (Table 3). Persistent iASD was observed in 58.6% of patients undergoing LAAO with 4D ICE and 50.0% of those utilizing 2D ICE, compared with 27.2% in cases performed without left atrial ICE (overall *p* = 0.001).

**Table 3:** Incidence of persistent iASD by intraprocedural imaging strategy

	4D ICE (*n* = 58)	2D ICE (*n* = 64)	No LA ICE (*n* = 81)	*P*-Value
Persistent iASD, *n* (%)	34 (58.6%)	32 (50.0%)	22 (27.2%)	0.001

Values are *n* (%). *P-*value represents overall comparison across groups using χ² test

When grouped together, cases involving left atrial ICE (2D or 4D) had a significantly higher incidence of persistent iASD compared with cases without left atrial ICE (54.1% vs. 27.2%, *p* < 0.001). There was no significant difference between 4D and 2D ICE techniques (*p* = 0.34).

### Multivariable logistic regression analysis

On multivariable logistic regression analysis (Table 4), both 2D and 4D left atrial ICE use were independently associated with increased odds of persistent iASD (2D ICE: OR 3.45, 95% CI 1.46–8.17, *p* = 0.001; 4D ICE: OR 3.57, 95% CI 1.52–8.38, *p* = 0.001), using no left atrial ICE as the reference group.

**Table 4:** Multivariable logistic regression analysis for independent predictors of persistent iASD

	Odds Ratio (95% CI)	*P*-Value
Intraprocedural Imaging Modality		
No LA ICE	1.00	
2D ICE	3.45 (1.46–8.17)	0.001
4D ICE	3.57 (1.52–8.38)	0.001
Baseline Characteristics		
Age	1.03 (0.98–1.09)	0.233
Male	0.69 (0.33–1.45)	0.321
Smoking	4.70 (0.75–29.37)	0.098
CHA_2_DS_2_-VASc ≥ 3	1.02 (0.19–5.38)	0.985
Heart Failure	1.65 (0.77–3.53)	0.200
Coronary Artery Disease	2.44 (1.12–5.28)	0.024
Cerebrovascular Accident	1.02 (0.43–2.39)	0.971
Diabetes Mellitus	1.49 (0.70–3.15)	0.303
Hypertension	0.41 (0.18–0.96)	0.040
Thyroid Disease	0.51 (0.22–1.20)	0.123
Cancer	1.36 (0.59–3.14)	0.465
Chronic Kidney Disease	0.84 (0.37–1.89)	0.666
Prior PVI	0.64 (0.21–2.01)	0.447
Left Ventricular Ejection Fraction	1.00 (0.96–1.03)	0.821
Procedural Characteristics		
LA Dwell Time	1.00 (0.98–1.01)	0.915
LAAO Device Size	0.99 (0.90–1.08)	0.763
Follow-up Characteristics		
Post-procedural Anticoagulation	0.95 (0.41–2.18)	0.901
Time to Follow-up Imaging	0.99 (0.98–1.01)	0.258

*PVI* pulmonary vein isolation, *LAAO*  left atrial appendage occlusion

Intraprocedural imaging modality modeled with no LA ICE as the reference group

Coronary artery disease was also independently associated with increased odds of persistent iASD (OR 2.44, 95% CI 1.12–5.28, *p* = 0.024), while hypertension was associated with decreased odds (OR 0.41, 95% CI 0.18–0.96, *p* = 0.040). No other baseline, procedural, or follow-up variables were significantly associated with persistent iASD.

## Discussion

In this single-center retrospective study, we found that persistent iASD was present on follow-up imaging in 43.3% of patients who underwent LAAO, and that use of intraprocedural left atrial ICE (2D or 4D) was associated with a higher incidence of persistent iASD compared to no left atrial ICE. However, there was no difference in incidence of persistent iASD when comparing procedures utilizing 2D ICE in the LA with those utilizing 4D ICE. Procedural characteristics, including left atrial dwell time and LAAO device size, were similar between patients with persistent iASD and those without. Major periprocedural complications were uncommon, and no significant association between persistent iASD and adverse clinical outcomes was observed in this cohort. However, the relatively small number of clinical events limits the ability to exclude modest differences between groups.

Importantly, these findings remained consistent after adjustment for baseline characteristics. On multivariable analysis, both 2D and 4D left atrial ICE use were independently associated with increased odds of persistent iASD, supporting the hypothesis that septal instrumentation plays a central role in defect persistence as opposed to differences in patient characteristics alone. In addition, coronary artery disease remained associated with increased odds of persistent iASD, while hypertension was associated with decreased odds, suggesting that underlying vascular and atrial remodeling may also influence septal healing.

Prior studies have reported variable rates of iASD persistence following transseptal procedures, likely reflecting differences in procedural technique, sheath size, and follow-up imaging modality [13, 15, 17]. The total incidence of persistent iASD reported in this study is toward the upper range of previously reported values. This may reflect the utilization of the transseptal ICE catheter in a majority of the procedures in this study, as the incidence of persistent iASD in the group without LA ICE was closer to the midpoint of values reported in prior studies (27.2%). This may also partially reflect the timing of follow-up imaging, as most prior studies began follow-up imaging around 90 days post-procedure, while in this study most follow-up imaging was done closer to 45 days after LAAO. Additionally, follow-up in this cohort was performed using both TEE and cardiac CT, modalities with high sensitivity for detection of residual interatrial shunting.

Although follow-up imaging modality differed between groups, there was no systematic institutional protocol dictating the choice of TEE versus cardiac CT, and selection was primarily based on operator preference and clinical circumstances. Importantly, TEE is generally considered more sensitive than cardiac CT for detection of small interatrial communications. Despite a lower proportion of TEE follow-up in the 4D ICE group, this cohort demonstrated the highest incidence of persistent iASD, suggesting that differences in follow-up imaging modality are unlikely to explain the observed association.

The present findings support the hypothesis that the additional septal instrumentation associated with LA ICE access may contribute to the persistence of iASDs. Single-puncture double transseptal LA access involves the sequential passage of multiple large-bore devices across the interatrial septum, which likely produces greater mechanical stretch and septal tissue deformation. This increased septal instrumentation provides a plausible explanation for the higher rate of persistent iASD observed in cases with LA ICE.

These findings also demonstrate no significant difference in iASD persistence between cases utilizing 4D ICE and 2D ICE. This indicates that the imaging modality is unlikely to be the primary driver of iASD persistence. Instead, the need for LA ICE catheter access and positioning, regardless of ICE catheter size, may represent the dominant procedural factor influencing healing of the atrial septum. Our analysis highlights the potential impact of septal instrumentation burden when selecting intraprocedural imaging strategies for LAAO.

The potential for increased iASD persistence should be considered in the context of the established advantages of ICE-guided procedures. Intracardiac echocardiography allows for procedural guidance without the need for a dedicated imaging operator and may facilitate avoidance of general anesthesia, which can be particularly advantageous in higher-risk patient populations. Comparative studies have generally demonstrated similar procedural success and overall complication rates with ICE- and TEE-guided LAAO. In one single-center study of 186 patients, Alkhouli et al. reported similar technical success and major procedure-related event rates between ICE- and TEE-guided procedures, while ICE was associated with shorter in-room time. Peri-device leak rates were also similar at follow-up [18]. In another large U.S. inpatient analysis, Zahid et al. found similar overall major complication rates between ICE-and TEE-guided procedures, although gastrointestinal bleeding was more common with TEE-guided procedures while retroperitoneal bleeding was more frequent with ICE-guided procedures [19]. However, any benefits of ICE guidance may be partially offset by the need for additional septal instrumentation when the ICE catheter is advanced into the left atrium. As the use of left atrial ICE expands, including for guidance of emerging technologies such as pulsed field ablation and for concomitant structural and electrophysiology procedures, the implications of persistent iASD may become increasingly relevant.

Despite the higher incidence of persistent iASD observed in LA ICE-guided procedures, no increase in adverse clinical events was observed in this cohort. Major periprocedural complications were rare overall, and persistent iASD was not associated with increased rates of bleeding, thromboembolic events or hospitalization during follow-up. Published meta-analyses comparing ICE- and TEE-guided LAAO have likewise demonstrated low absolute rates of major adverse events, no significant differences in cardiac tamponade, device embolization or stroke between imaging modalities [20]. Our current findings are therefore consistent with prior studies, most of which suggest that many iASDs have little to no observable hemodynamic or clinical impact [15]. Although no increase in clinical events was observed in this cohort, persistent interatrial communication raises the theoretical risk of paradoxical embolism. This may be particularly relevant in patients undergoing LAAO, many of whom have contraindications to long-term anticoagulation, and therefore may be at higher risk for thromboembolic complications in the setting of right-to-left shunting. Ultimately, the clinical significance of persistent iASD after LAAO remains incompletely defined, particularly over longer-term follow-up and in higher risk subgroups. Therefore, the long-term implications of iASD warrant further investigation.

This study builds upon prior observations regarding iASD persistence after transseptal interventions by specifically examining current LAAO techniques which incorporate ICE guidance via single-puncture double transseptal LA access. Previous studies examining both structural and electrophysiology procedures have demonstrated that larger sheath sizes and multiple septal crossings are associated with increased rates of persistent defects. More specifically, in 2017 Korsholm et al. found that residual iASD was detected in 34.7% of patients who underwent ICE-guided LAAO and 25.8% of those who underwent TEE-guided LAAO, with follow-up imaging performed at a median of 55 days post-procedure [21]. However, this result was not statistically significant, and the procedures in the study involved only 2D ICE catheters. Therefore, our study is directionally consistent with prior literature and further suggests that single-puncture double transseptal access strategies used during ICE-guided LAAO may similarly diminish septal integrity. Importantly, the current analysis also demonstrates comparable iASD persistence rates between procedures involving 4D ICE and 2D ICE, providing novel insight into the relative contributions of imaging modality versus septal instrumentation.

### Limitations

This study has several limitations. First, the fact that the study was single-center limits generalizability, and the retrospective design introduces the potential for selection bias. Second, timing of follow-up imaging was not fully standardized, which may have influenced the detection of small residual septal defects. Third, readers were not blinded to LAAO procedural strategy, which may introduce ascertainment bias. Fourth, because this was a retrospective observational study including all consecutive eligible patients during the study period, no power calculation was performed in advance. The number of clinical outcome events was low, which may have limited statistical power to detect differences in relatively infrequent clinical endpoints. Accordingly, larger studies will be necessary to more definitively evaluate whether procedural imaging strategy or persistent iASD influences clinical outcomes. Fifth, the follow-up imaging modality differed between procedural groups. Although TEE is generally considered more sensitive than cardiac CT for detection of small interatrial communications, the 4D ICE group had the lowest proportion of TEE follow-up despite demonstrating the highest incidence of persistent iASD. Nevertheless, differences in follow-up imaging modality may have influenced rates of iASD detection.

Finally, long term follow up was not available for most patients, so we were unable to draw conclusions regarding the natural history of persistent iASD beyond the early post-procedural period. Because many iASDs identified during the early post-procedural period undergo spontaneous closure over time, we were unable to determine the long-term natural history of these defects or whether the differences observed between imaging strategies persist beyond early follow-up.

Despite these limitations, this study represents one of the largest contemporary cohorts evaluating persistent iASD following LAAO, and introduces a novel comparison between procedural groups using 2D and 4D ICE in the left atrium.

## Conclusion

Persistent iatrogenic atrial septal defect is common following LAAO, occurring in approximately 43% of patients at early follow-up. Use of intracardiac echocardiography with left atrial access is independently associated with a higher incidence of persistent iASD, whereas rates are similar between 4D and 2D ICE techniques. Although no significant association between persistent iASD and adverse clinical outcomes was observed in this cohort, the long-term clinical significance of persistent iASD after contemporary LAAO warrants further investigation.

## Data Availability

Raw data available from study authors upon request.

## Declarations

### Capsule summary

Persistent iatrogenic atrial septal defect (iASD) in 43% of patients after left atrial appendage occlusion and occurred more frequently in cases utilizing left atrial intracardiac echocardiography (ICE). Rates were similar between groups involving 2D and 4D ICE, suggesting that septal instrumentation rather than imaging modality drives iASD persistence.
